# Supplementary material for: Optical detection of single sub-15 nm objects using elastic scattering strong coupling
Source: Nat Commun. 2025 Aug 29;16:8101. doi: 10.1038/s41467-025-63380-8 (PMC12397264; doi:10.1038/s41467-025-63380-8)
Supplement: Supplementary file 1 — Supplementary Information [file 41467_2025_63380_MOESM1_ESM.pdf]

Supplementary information optical detection of single sub-15 nm objects using elastic scattering strong coupling

MohammadReza Aghdaee[1], Melissa J. Goodwin[2], Oluwafemi S. Ojambati[1*]

[1]Faculty of Science and Technology, MESA+ Institute for Nanotechnology, University of Twente, Enschede, The Netherlands.
[2]Nanolab, MESA+ Institute of Nanotechnology, University of Twente, Enschede, The Netherlands.

*Corresponding author(s). E-mail(s): o.s.ojambati@utwente.nl;

SI. A Scattering and absorption cross-sections

In the main text, we used the extinction cross-section to align our simulation with experimental results, as our experimental setup measures the extinction spectrum directly. However, the absorption and the scattering cross-sections also show similar modes (Supplementary Fig. 1). We simulate an Au nano-object inside a nanocavity for different nano-object diameters, and also the empty nanocavity with a nanoprobe diameter of 90 nm. There are similar modes in all the cross-sections for both the nanocavity with a nano-object inside the gap and the empty nanocavity. The mode in the cross-section of the empty nanocavity corresponds to the nanocavity mode [1–3]. While we describe the origin of the nano-object-in-cavity modes in the main text, the nanocavity mode of the empty cavity can be understood from the perspective of a metal-insulator-metal (MIM) system. A plasmonic nanocavity can be approximated as an MIM structure due to the flat facet of the nanoprobe and dielectric gap on the Au film. By applying boundary conditions at the two metal-dielectric interfaces, one obtains a dispersion relation that defines the resonance conditions, such as Fabry-Perot-like modes [1, 2]. The dominant resonance in the optical cross-section spectra of the empty nanocavity arises from the confinement of electromagnetic fields within the plasmonic gap [3]. This nanocavity mode redshifts with increasing nanoprobe diameter (Supplementary Fig. 1f, Fig. 1d), and blueshifts with increasing gap size

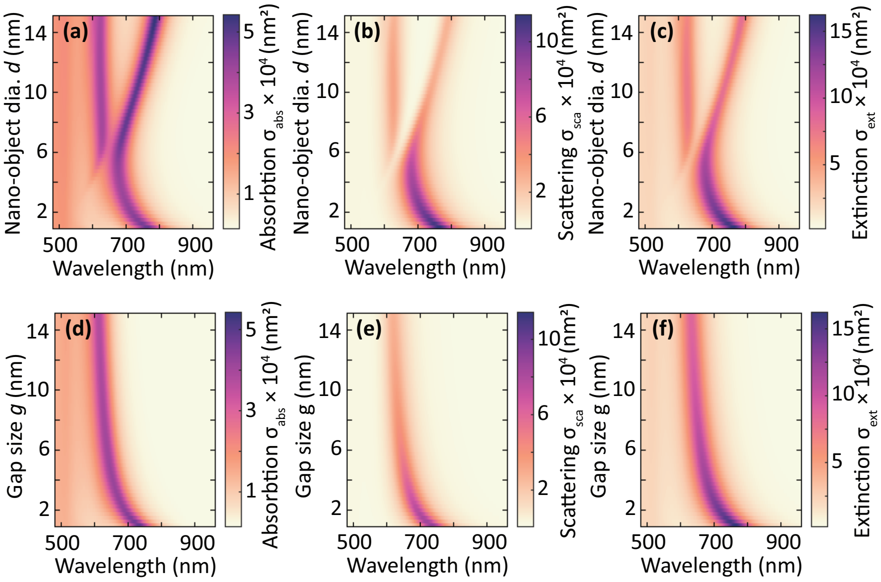

Supplementary Fig. 1 **(a)** Absorption cross-section (σ_{abs}), **(b)** scattering cross-section (σ_{sca}), **(c)** extinction cross-section (σ_{ext}) spectra of an Au nano-object inside nanocavity for different nano-object diameters. **(d)** Absorption cross-section (σ_{abs}), **(e)** scattering cross-section (σ_{sca}), **(f)** extinction cross-section (σ_{ext}) spectra of an empty nanocavity for different gap sizes. (a-f) The nanoprobe diameter is 90 nm.

(Supplementary Figs. 1d-f), in agreement with MIM-based theoretical predictions and previous reports [2].

SI. B Effect of excitation polarization

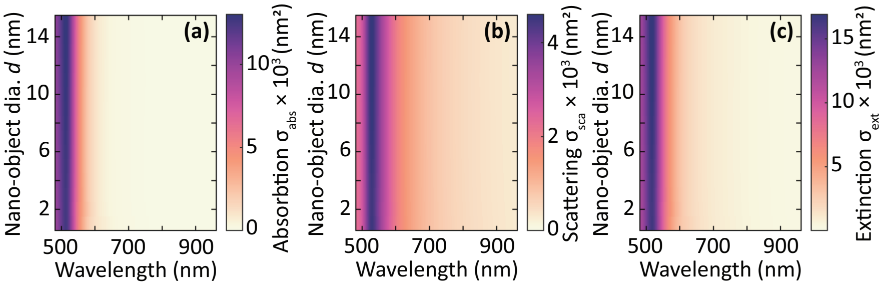

Supplementary Fig. 2 TE polarized excitation. Absorption cross-section (σ_{abs}), **(b)** scattering cross-section (σ_{sca}), **(c)** extinction cross-section (σ_{ext}) spectra of an Au nano-object inside nanocavity for different nano-object diameters. The nanoprobe diameter is 80 nm.

The polarization of the excitation can excite different nano-object-in-cavity modes. Transverse magnetic (TM) polarized light excites the nanocavity mode where the two

resonators, nano-object and nanocavity, can couple to each other. When the structure is excited using a transverse electric (TE) polarized light, due to the absence of the z component of the electric field, only the transverse mode of the nanoprobe is excited, which dominates the total cross-sections (Supplementary Fig. 2). The absence of nano-objet-in-cavity modes is because the nanoprobe and gold film are not coupled as when using a TM-polarized light [4].

SI. C Sensitivity of nano-object-in-cavity modes

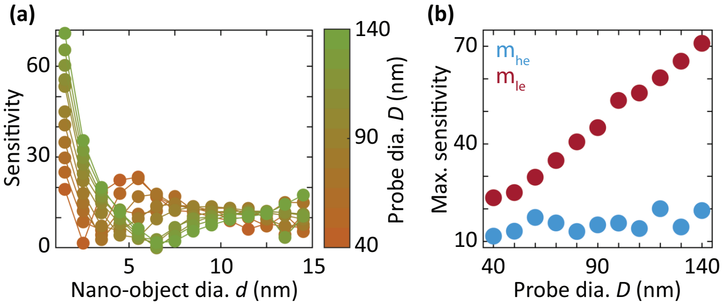

Supplementary Fig. 3 **(a)** The sensitivity of the lower energy nano-object-in-cavity mode, which is defined as the changes in the resonance wavelength over the changes in the diameter of the nano-object ($\frac{\partial \lambda}{\partial d}$). **(b)** The maximum sensitivity as a function of probe diameter for lower energy mode ($\mathrm{m_{le}}$, red markers) and higher energy mode ($\mathrm{m_{he}}$, blue markers).

There are two modes present in the cross-sections of the nano-object in the nanocavity, which are sensitive to the size and material of the nano-object. We quantify the sensitivity ($\frac{\partial \lambda_\mathrm{m}}{\partial d}$) of our technique from extinction cross-section. We notice nonlinear wavelength changes to small changes in nano-object diameter (Supplementary Fig. 3a), which is consistent for different diameters of the nanoprobes from 40 nm to 140 nm. We extract the maximum sensitivity for all nanoprobe diameters, which range from 23 to 71 and 12 to 20 for lower energy mode ($\mathrm{m_{le}}$) and higher energy mode ($\mathrm{m_{he}}$), respectively (Supplementary Fig. 3b). Overall, the lower energy mode shows a stronger dependence on nano-object diameter compared to the higher energy mode. Also, as the diameter of the nanoprobe increases, the maximum sensitivity of the lower energy mode increases. Several studies have explored methods to detect small nano-objects, and we have summarized the maximum sensitivity achieved in Supplementary Table 1. In contrast with existing techniques, the presence of a nano-object induces a resonance shift within the nanocavity, offering enhanced sensitivity compared to other platforms that rely on resonance shifts in nanophotonic structures for detection.

Supplementary Table 1 Maximum sensitivity of nano-object diameter

Maximum sensitivity ($\frac{\partial \lambda}{\partial d}$)	0.004	0.006	<0.001	<0.001	0.001	71
Reference	[5]	[6]	[7]	[8]	[9]	This work

SI. D Sample characterization

To verify the sample fabrication, we perform AFM measurements on Au film and on two other samples: a nanocavity with a nano-object inside the gap and a nanoprobe directly on the Au film (Supplementary Figs. 4a-c). The nanoprobe size is 80 nm for both samples, and the nano-object diameter is 12 nm (Supplementary Figs. 4a,b). There are several large structures ($>$ 500 nm) in the sample with the nano-object inside the gap due to the aggregation of nanoparticles. The scattering of such large structures is significantly higher and are avoided in the dark-field measurements. We analyze nanoparticles from both samples (Supplementary Figs. 4d,e). The measured nanocavities show a narrow height variation of about 6 nm for both samples, which is also consistent with the reported diameter variation of 6 nm from the manufacturer (Supplementary Fig. 4f).

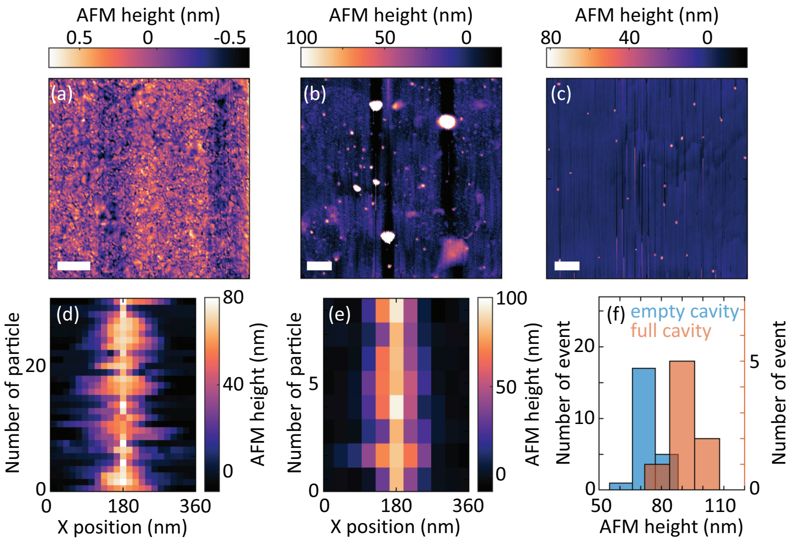

Supplementary Fig. 4 The AFM measurements. **(a)** Template striped Au film. The scale bar is 500 nm. **(b)** Nanoprobes directly on the Au film and **(c)** a nanocavity with a nano-object inside the gap. The scale bar is 2 μm. Radial profile of the nanoparticles on a sample with **(d)** nanoprobes directly on the Au film and **(e)** nanocavities with a nano-object inside the gap. **(f)** The histogram of the nanoparticle heights on the samples in (d) and (e). The diameter of the probe is 80 nm, and the nano-object diameter is 12 nm.

We use a scanning electron microscope (SEM) and focused ion beam lift-out technique to prepare samples for SEM cross-sectional images. The surface of the sample is coated with a carbon layer as a protective layer for the cross-sectioning process. The surface is imaged, and then a nanoprobe is selected to obtain the cross-sectional images. A focused ion beam mill creates a hole around the nanoparticle region, isolating the nanoparticle from its surroundings. The isolated region is lifted out using a lamella and mounted onto a grid. The sample is further thinned and polished to capture

SEM cross-sectional images. The diameter of the nano-objects is 12 nm (Supplementary Fig. 5). Unfortunately, due to the fragile nature of the compound nanostructure, cross-sectional imaging is extremely challenging. Accurate cross-sectioning requires precise targeting of the region directly beneath the nanoprobe—where the nano-object resides—yet our FIB system is constrained by a 100 nm step size in the y-direction (Supplementary Fig. 5a). Consequently, most FIB cross-sections did not directly capture the nano-object within the gap. In such cases, SEM imaging often revealed the nanoprobe at a height consistent with the diameter of the nano-object, supporting the presence of the nano-object beneath it (Supplementary Figs. 5b,c). Despite these limitations, Supplementary Fig. 5c shows an SEM image where the nanoprobe is assembled on top of the nano-object, lending further support to our proposed structural model.

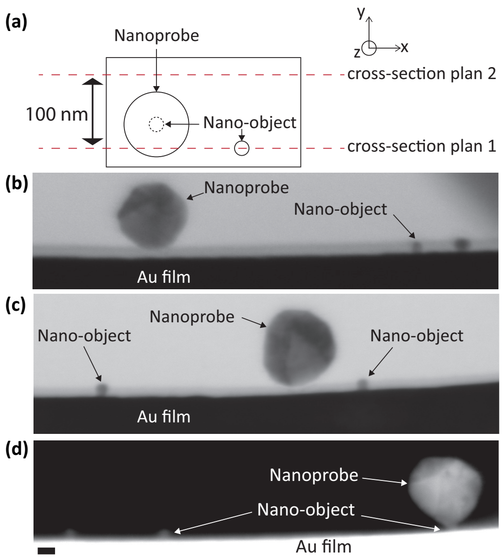

Supplementary Fig. 5 The SEM cross-sectioning. **(a)** The schematic of the SEM cross-sectional processes; **(b,c)** Bright-field image of a nanoprobe, the nano-objects on the sides are visible; **(d)** Two individual nano-objects sitting on the Au film on the left and a nanocavity with a nano-object inside the gap on the right. The scale bar is 10 nm.

Nano-object size distributions were extracted from SEM images using a custom-made MATLAB script. Each SEM image was binarized, and individual particles were identified via connected component analysis and marked for later analysis (Supplementary Fig. 6a). A pixel-to-length conversion factor extracted from the image metadata was applied to enable accurate dimensional analysis. Assuming a circular nano-object, the diameters were calculated from the detected areas. Histograms of the resulting diameters were then generated, and Gaussian functions were fitted to quantify the distributions using the standard deviation of the fitting (Supplementary Fig. 6b). These size distributions were subsequently used in the analysis presented in Fig. 3d. The SEM analysis reveals a measurement uncertainty averaging below 1 nm, which is in agreement with the error obtained from the manufacturer. For nano-objects within

this sub-15 nm range, a polydispersity index of 20–25% is commonly accepted as a typical variation in size distribution.

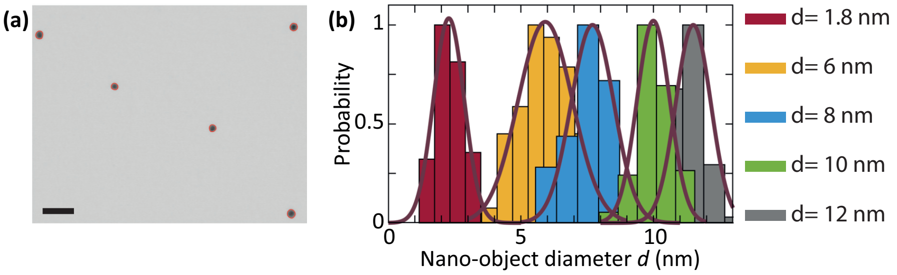

Supplementary Fig. 6 **(a)** SEM image showing nano-objects with detected regions highlighted in red. These marked regions are used for quantitative analysis to determine the diameters of the individual nano-objects. The scale bar is 50 nm. **(b)** Normalized histograms of nano-object diameter distributions. Each histogram is color-coded by size and fitted with a Gaussian function (solid lines).

SI. E Experimental setup

We use a custom-made dark-field microspectrometer to capture the dark-field images and measure the dark-field scattering of the nanocavity. The experimental setup is shown in the Supplementary Fig. 7. A near-infrared enhanced halogen lamp (Thorlabs OSL2BIR) illuminates the sample with a dark-field objective (Olympus MPlanFL N 100x/0.90 NA) at an incident angle larger than 65°. The scattered light is collected using the same objective and split into two paths for dark-field imaging and spectroscopy. We use a beam splitter (Thorlabs BSW26) that has a coating for the 350 - 1100 nm range. A spectrograph (Andor Shamrock 500i) has a 300 lines/mm grating, with an EMCCD camera (Andor Newton 970) providing ≈ 160 nm wavelength range. We capture the spectrum from 400 nm to 1050 nm by concatenating 5 spectra. The acquisition time is 1 second, and to increase the signal-to-noise ratio, we average over 10 spectra.

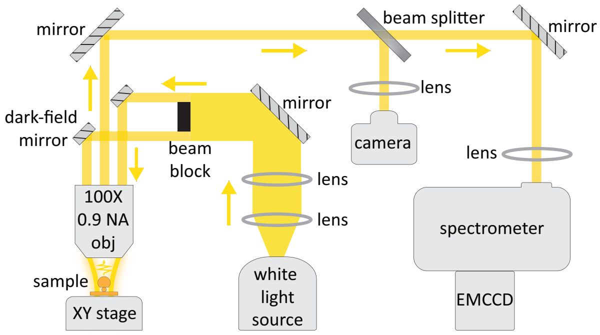

Supplementary Fig. 7 Custom-made dark-field micro-spectrometer used to measure the dark-field scattering of the nanocavities and capture the dark-field images.

SI. F Effect of the surface density of the nano-objects

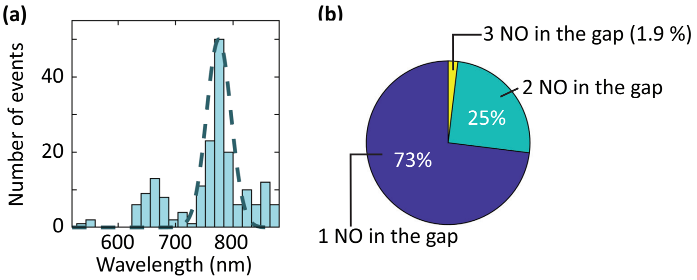

Supplementary Fig. 8 **(a)** The histogram of the resonance peak of the measured nanocavities with a 2.2 nm nano-object inside the nanocavity with an 80 nm nanoprobe. The density of nano-objects is 640000 particles/μm^2. The dashed line represents a Gaussian fit to the dominant detected maxima in the histogram. **(b)** Percentage distribution of the number of nano-objects present inside the nanocavity. The majority of cavities contain one nano-object (73%), followed by two nano-objects (25%), and a small fraction with three nano-objects (1.9%).

The resonance wavelengths of Au nano-objects with a diameter of 1.8 nm to 12 nm inside a nanocavity are shown in Fig. 3d. We note that there is a weak peak with a lower occurrence frequency at around 780 nm for several nano-object diameters. As a control measurement, we measure a sample where there is more than one nano-object inside the nanocavity. We use a high particle density of 640000/μm^2. This results in forming nanocavities with several nano-objects inside the gap. The excessive nano-objects can form multilayers or are aggregated. The nano-object diameter is 2.2 nm, and the probe diameter is 80 nm. The dark-field measurements results show a dominant mode at around 780 nm (Supplementary Fig. 8). This mode is similar to the lower occurrence mode present in Fig. 3a. However, the occurrence frequency of the mode observed with a higher surface density is higher compared to one with a lower surface density. We attribute this mode to the presence of more than one nano-object in the nanocavity.

The average spacing between nano-objects is 25 nm (Fig. 2b), and there is inherent variation in their distribution. To quantify the probability of having multiple nano-objects within a nanocavity, we performed a Monte Carlo simulation based on the experimentally derived distribution (Fig. 2b). Out of 100,000 trials simulating random nanoprobe placement, approximately 73% of nanocavities contained one nano-object, 25% contained two, and 2% had three. This result indicates that while single occupancy is dominant, multiple-object scenarios are statistically probable. These results are consistent with our dark-field measurements, where the frequency of the peak attributed to 2 or more nano-objects in the cavity is 18% for 2.2 nm, 22% for 8 nm, and 19% for 10 nm nano-objects.

SI. G Effect of the tilted nanoprobe

In our simulations, we assumed that the facet of the nanoprobe remains parallel to the Au film. To fabricate the nanocavity with a nano-object inside the gap, the nanoprobe was drop-cast to form the cavity. While this method could potentially introduce a tilt in the nanoprobe, cross-sectional SEM images (Supplementary Fig. 5) confirm that the probes have a tilt angle close to 0°, supporting the assumption of uniform spacing within the gap. To evaluate the influence of possible tilt, we performed numerical simulations (Supplementary Fig. 9) using the maximum angle at which the probe is in contact with the Au film. The extinction spectra indicate that probe tilt induces significant perturbations in the cavity resonance modes. The agreement between the experimentally measured dark-field spectra and simulations assuming a 0° tilt further supports that the nanoprobe is aligned with the nano-object and Au film.

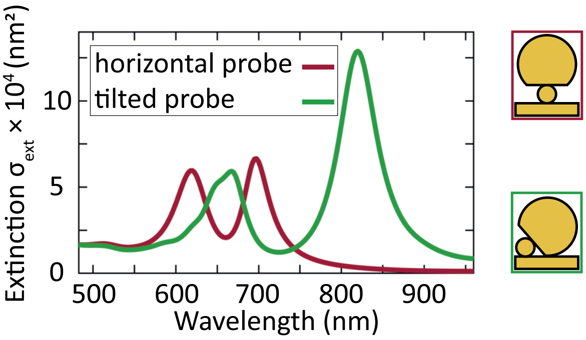

Supplementary Fig. 9 Extinction cross-section (σ_{ext}) spectra of a 7 nm Au nano-object inside a nanocavity **(a)** nanoprobe has 0° tilt, **(b)** nanoprobe has 32° tilt. The nanoprobe diameter is 80 nm.

SI. H Effect of nano-object position

In the experiment, the lateral position of the nano-object inside the nanocavity is not controlled. However, the variation of the lateral position of the nano-object in the gap can result in a change in the nano-object-in-cavity resonance modes. The calculated extinction spectra of a 5 nm gold nano-object with a 40 nm nanoprobe for different x positions show that two modes consistently appear from x = 0 nm to x = 6.5 nm (Supplementary Fig. 10). However, when the center of the nano-object reaches the facet of the nanoprobe, both modes gradually blueshift, and eventually, the higher energy mode disappears. For x > 8 nm, only one resonance mode in the extinction spectrum appears at 575 nm, which is the same as the resonance of an empty nanocavity.

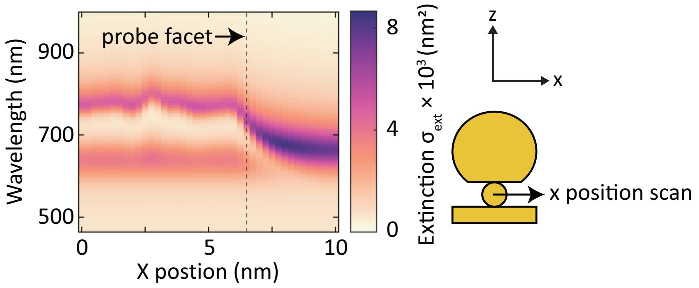

Supplementary Fig. 10 Extinction cross-section (σ_{ext}) spectra of a 5 nm Au nano-object inside nanocavity for different x position. The nanoprobe diameter is 40 nm.

SI. I Modeling the resonance of the nano-object in the nanocavity

In the presence of the nanoprobe and Au film, the resonance energy of the nano-object is altered compared to an isolated nano-object. We start with simulating a nano-object close to the Au film (Supplementary Fig. 11a). There are two resonance modes in the scattering cross-section for a nano-object on top of the Au film: a higher energy mode and a lower energy mode, which is due to the coupling of the nano-object to its image charge in the Au film. The presence of the nanoprobe on top further perturbs the resonance of the nano-object on the Au film. We use the lower energy mode of the nano-object on the film (E_{noom}) as the resonance energy of the uncoupled nano-object and include the perturbation of the nanoprobe on top. Therefore $E_{\text{no}} = \text{A}E_{\text{noom}}$, where A accounts for the perturbation of resonance energy due to nanoprobe. From fitting the nano-object-in-cavity modes to Eq. 1 the extracted $\text{A} = 0.84$ to 0.89 for $d = 4$ nm to $d = 11$ nm. The resonance of the nano-object in the

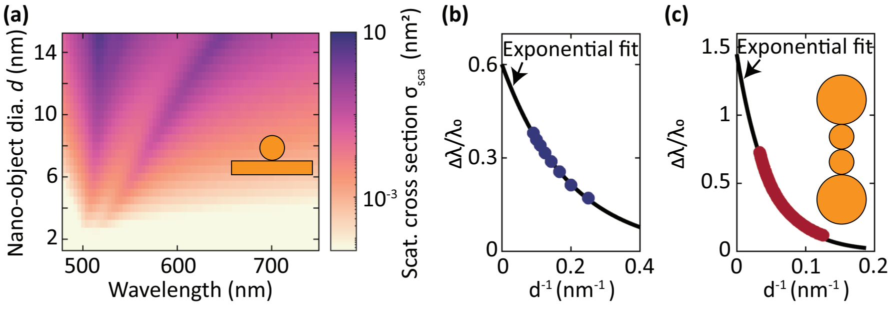

Supplementary Fig. 11 **(a)** Scattering cross-section (σ_{sca}) spectra of an Au nano-object on Au film for different nano-object diameters. **(b)** The resonance of the nano-object in the nanocavity environment extracted from fitting the nano-object-in-cavity modes to Eq. 1 (marker) and a fitted exponential function ($ae^{-x/b}, a = 0.6, b = 0.2, x = 1/d$, line). **(c)** The resonance of the nano-object in the nanocavity environment extracted from the dipolar coupling model (marker) and a fitted exponential function ($ae^{-x/b}, a = 1.44, b = 0.05, x = 1/d$, line)

nanocavity changes exponentially with the diameter of the nano-object (Supplementary Fig. 11b). To explain the resonance of the nano-object inside the nanocavity, we employ a simple dipolar coupling model. In the case of a simple nanoparticle dimer, the dipolar coupling approach models the total electric field felt by each nanoparticle to be proportional to the polarizability of the structure, where the maximum polarizability can be extracted to determine the resonance of the structure [10–12]. The dipolar coupling approach is generalized and tested for complex nanostructure geometries, including three nanoparticles in a linear arrangement [13].

We model the perturbed resonance of the nano-object inside the nanocavity based on the dipolar coupling model and include the non-local effect of the small nano-object. We first model the nano-object on the Au mirror and then include the effect of the nanoprobe and its image charge. For a single nanoparticle in a medium with the dielectric function of ε, the induced electric dipole moment μ in an external electric field E is given as [14]:

$$\mu = \alpha \varepsilon E, \tag{1}$$

where α is the polarizability of the nanoparticle, and it follows $\alpha_{\text{no}}^{\text{nl}}$ from Eq. 2 in the Methods section for small nano-objects. The polarizability reduces to $\alpha_{\text{np}} = \frac{\pi}{2} D^3 \frac{\varepsilon_m - \varepsilon}{\varepsilon_m + 2\varepsilon}$ for nanoprobe, due to the large diameter of the nanoprobe. The nano-object on the mirror can be approximated to a nano-object dimer due to the coupling of the nano-object to its image charge in the Au mirror [15]. Based on the dipolar coupling model, the total electric field (E_{dimer}) felt by the nano-object in the presence of neighboring image charge is the summation of the incident light field E and the near-field of the electric dipole on the image charge [10]:

$$E_{\text{dimer}} = E + \frac{K \mu_{\text{no}}}{\pi \varepsilon (d+s)^3}, \tag{2}$$

where μ_{no} is the induced dipole moment of the nano-object image charge in the Au film, s is the spacing between the nanoparticles, and K is an orientation parameter and depends on the alignment of the nanoparticle dipoles. The constant $K = 2$ for dipoles arranged head-to-tail corresponding to in axis polarization [16, 17]. Considering Eq. 1 and Eq. 2, the net polarizability of the nano-object dimer is given as:

$$\alpha_{\text{dimer}} = \frac{\alpha_{\text{no}}^{\text{nl}}}{1 - \frac{K \alpha_{\text{no}}^{\text{nl}}}{\pi (d+s)^3}}. \tag{3}$$

To account for the effect of the nanoprobe and its image charge, we include their near-field electric dipole in the total electric field (E_{t}). The factor of 2 arises due to the contribution of the image charge of the nanoprobe in the Au film, enhancing the interaction. Therefore, the total electric field felt by the nano-object inside the nanocavity is given by:

$$E_{\text{t}} = E_{\text{dimer}} + \frac{2K \mu_{\text{np}}}{\pi \varepsilon (d + \frac{D+3s}{2})^3}. \tag{4}$$

Similar to Eq. 3, considering Eq. 1 and Eq. 4, the net polarizability of the combined system is given as:

$$\alpha_{\mathrm{t}} = \frac{\alpha_{\mathrm{dimer}}}{1 - \frac{2K\alpha_{\mathrm{np}}}{\pi(d+\frac{D+3s}{2})^3}}. \tag{5}$$

The scattering cross-section

$$\sigma_{\mathrm{sca}} = \frac{1}{6\pi}(\frac{2\pi}{\lambda})^4\alpha_{\mathrm{t}}^2 \tag{6}$$

is proportional to the polarizability, and we extract the resonance energy (λ_{res}) from the scattering cross-section [18]. The normalized resonance energy fits an exponential decay ($ae^{-x/b}, a = 1.44, b = 0.05, x = 1/d$, line, Supplementary Fig. 11c). The resonance energy is normalized to the one of an isolated nano-object ($\Delta\lambda = \lambda_{\mathrm{res}} - \lambda_0$, where λ_0 is the resonance of the isolated nano-object). We note that the fitting parameters a and b of exponential fit are different for experimental data compared to the model shown in Supplementary Fig. 11b. This difference is due to the dipolar model underestimating the coupling strength at small inter-particle gaps [10]. However, the dipolar coupling model qualitatively explains the exponential decay of the resonance energy of the nano-object inside the nanocavity as the diameter of the nano-object increases.

SI. J Strong coupling criteria

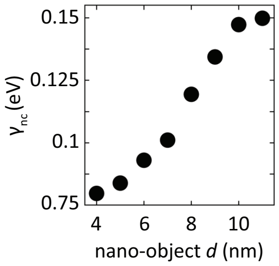

Supplementary Fig. 12 The bandwidth γ_{nc} of the nanocavity resonance mode.

Coupling is typically classified into weak, moderate, and strong regimes. The interaction between the resonator determines the strength of the coupling. Weak coupling happens when the losses of both resonators—the empty nanocavity and the nano-object on the Au film—overcome the coupling strength, resulting in enhanced emission but no spectral splitting. Moderate coupling occurs when the coupling strength becomes comparable to the combined losses, leading to partial hybridization of modes and the onset of spectral modifications such as broadening or asymmetry. In the strong coupling regime, the coupling strength exceeds the losses of both resonators, enabling coherent energy exchange between light and matter, which manifests as clear Rabi

splitting and the formation of hybrid states. To determine that the coupling of our system falls in the strong coupling regime, we extract γ_{nc}, and γ_{no}. Rabi splitting E_{Rabi} is defined as the energy difference between the coupled modes at the avoided crossing, giving $E_{\mathrm{Rabi}} = \sqrt{(\hbar\Omega_{\mathrm{R}})^2 - \left(\frac{\gamma_{\mathrm{no}}-\gamma_{\mathrm{nc}}}{2}\right)^2}$ [19]. The simulation results and strong coupling fitting results yielded values for $\hbar\Omega_{\mathrm{R}} = 0.13$ eV to 0.23 eV, $\gamma_{\mathrm{no}} = 0.04$ eV, and $\gamma_{\mathrm{nc}} = 0.08$ eV to 0.15 eV (Supplementary Fig. 12). The difference between γ_{no} and γ_{nc} is due to the larger size of the nanoprobe compared to the nano-object [20]. We obtain $E_{\mathrm{Rabi}} = 0.12$ eV to 0.22 eV for nano-objects with a diameter of 4 nm to 11 nm, respectively. This E_{Rabi} indicates that our hybrid nano-object inside the nanocavity system is above the onset of the strong coupling regime according to the criterion: $E_{\mathrm{Rabi}} > \frac{\gamma_{\mathrm{nc}}+\gamma_{\mathrm{no}}}{2}$. Nano-objects composed of several metals are also employed in nanocavities. We extract the γ_{no} values for Au, Ag, Cu, and Al nano-objects as 0.04 eV, 0.03 eV, 0.03 eV, and 0.085 eV, respectively. The obtained Rabi splittings overcome the losses and demonstrate that our system is above the onset of the strong coupling regime for different materials.

SI. K Tuning the nano-cavity resonance

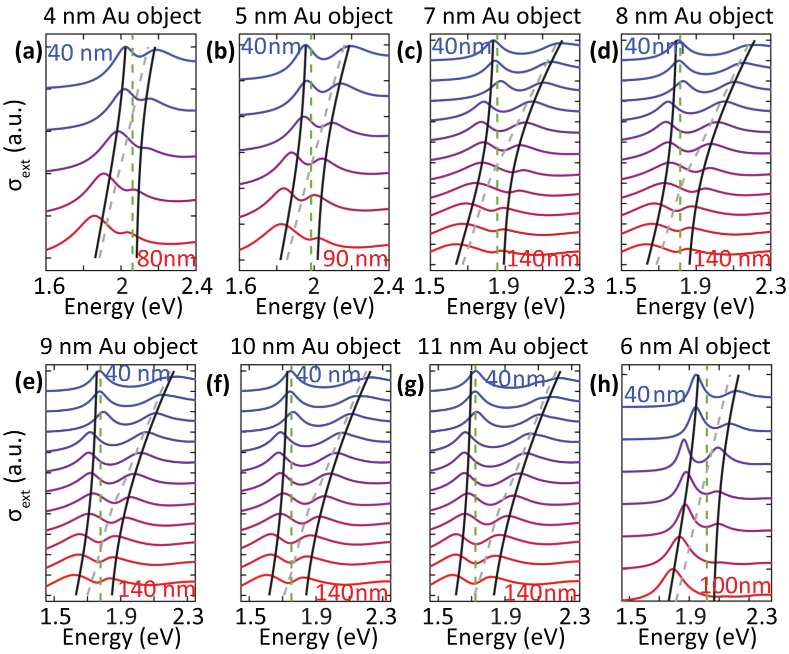

Supplementary Fig. 13 Normalized extinction cross-section (σ_{ext}) spectra of a Au nano-object inside nanocavity for different nano-object diameters ranging from 4 nm to 11 nm (a-g). Extinction cross-section (σ_{ext}) spectra of a 6 nm Al nano-objects (h). The black lines indicated the anti-crossing of the resonance modes derived from two strongly coupled resonator model. The resonance of the nano-object on the Au film is the green dashed line, and that of the nanoprobe on the film is the gray dashed line.

We investigate the origin of the nano-object-in-cavity modes by modeling the interaction between the nano-object and the plasmonic nanocavity with two coupled oscillators model. We tune the resonance of the nanocavity by adjusting the nanoprobe diameter from 40 nm to 140 nm. Furthermore, we investigate the effect of nano-object diameter and material. We once used Au nano-object and scanned the nano-object diameters from 4 nm to 11 nm (Supplementary Figs. 13a-g). Then, we keep the nano-object diameter fixed at 6 nm and use nano-objects with different materials, including Au, Ag, Cu, and Al (Supplementary Fig. 13g, Figs. 3e, 4a). Note that the anti-crossing of the resonance modes consistently appears for different nano-objects as the nanocavity resonance is tuned. We extract the Rabi splitting by fitting the nano-object-in-cavity modes to the two strongly coupled resonators model for each nano-object diameter and different material (Figs. 3f, 4b).

To experimentally investigate that the nano-object strongly couples to the nanocavity, we tune the resonance of the nanocavity using 60 nm, 80nm, 100 nm, and 125 nm probe diameters (Supplementary Fig. 14). There are two dominant resonances for different probe diameters, discussed in the main text. There is a more pronounced transverse mode present in the scattering histogram for larger probe diameters of 100 nm and 125 nm at around 530 nm.

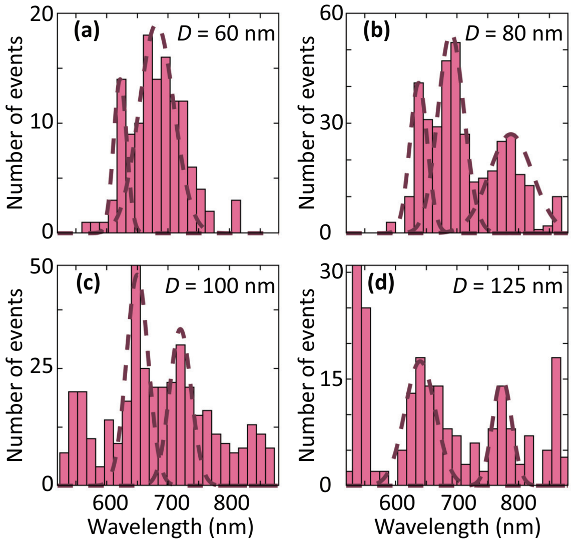

Supplementary Fig. 14 The histogram of the scattering peak of 6 nm Au nano-object inside nanocavities with a **(a)** 60 nm, **(b)** 80 nm, **(c)** 100 nm, and **(d)** 125 nm nanoprobe. The dashed line is a Gaussian fit to the detected maxima in the histogram.

SI. L Scattering of metallic nano-objects and coupling strength

We use the hydrodynamic model to account for the nonlocality effect of the small metallic nano-objects. In this model, we use the permittivity of the metal from the Drude model introduced in the non-local hydrodynamic model. The parameters for permittivity of different metals are taken from the literature [3, 21–26] (Supplementary Table. 2). The coupling strength for two strongly coupled resonators is given by [27]:

Supplementary Table 2 Parameters in the Drude model for different metals

	Al	Ag	Au	Cu
ω_p	12.04	8.9	8.37	6.79
ε_∞	1	4.5	10.5	6.56
γ	0.128	0.0588	0.069	0.061

$$\Omega_{\mathrm{R}} = \mu_{\mathrm{m}} \sqrt{\frac{4\pi n c}{\hbar \lambda \varepsilon V_{\mathrm{m}}}}. \tag{7}$$

Here μ_{m} is the transition dipole moment of isolated nano-object, $\hbar$ is Planck constant, c is the speed of light traveling through a vacuum, n is the number of atoms and V_{m} is the mode volume. The plasma frequency of metals is given by [28]:

$$\omega_{\mathrm{p}}^2 = \frac{4\pi N e^2}{m}. \tag{8}$$

Where e is the charge of the electron, m is the effective mass of the electron, and N is the number density of the electrons in the nano-object. Using Eq. 7 and Eq. 8 and considering $N = n/V_{\mathrm{n}}$, we obtain:

$$\Omega_{\mathrm{R}} = \omega_{\mathrm{p}} \frac{\mu_{\mathrm{m}}}{e} \sqrt{\frac{c m V_{\mathrm{n}}}{\hbar \lambda \varepsilon V_{\mathrm{m}}}} \tag{9}$$

a linear relationship between the coupling strength and the plasma frequency.

In the main text (Non-local hydrodynamic model), we introduced the dielectric function of metals $\varepsilon_{\mathrm{m}}(\omega)$, which can be rewritten in two real and imaginary parts as [28]:

$$\varepsilon_m(\omega) = \varepsilon_\infty - \frac{\omega_p^2}{\omega^2 + \Gamma^2} + i \frac{\omega_p^2 \Gamma}{\omega\left(\omega^2 + \Gamma^2\right)}. \tag{10}$$

Eq. 10 implies that both the real and imaginary parts of the dielectric function are functions of the plasma frequency. Therefore, changes in the real part or imaginary part of the dielectric function directly impact the Rabi splitting and nano-object-in-cavity modes.

One can write Eq. 7 as a function of the diameter of the nano-object. The number of nano-object atoms contributed in the plasmonic coupling can be estimated using the Au atom density N_n as:

$$\mathrm{n} = \frac{4}{3}\pi N_\mathrm{n}(\frac{d}{2r_0})^3. \tag{11}$$

Also, we approximate the nanocavity mode volume using the geometry of the nanogap as:

$$V_\mathrm{m} = \pi(\frac{3}{8}\frac{D}{2})^2 d, \tag{12}$$

due to the confinement of the mode in the cylindrical gap with a radius of the nanoprobe facet and the gap size to be the same as the diameter of the nano-object. Therefore the Eq. 7 is simplified as:

$$\Omega_\mathrm{R} = \frac{8}{3}\mu_\mathrm{m}\frac{d}{D}\sqrt{\frac{2\pi c N_\mathrm{n}}{3\hbar\lambda\varepsilon r_0}}. \tag{13}$$

SI. M Electric field localization

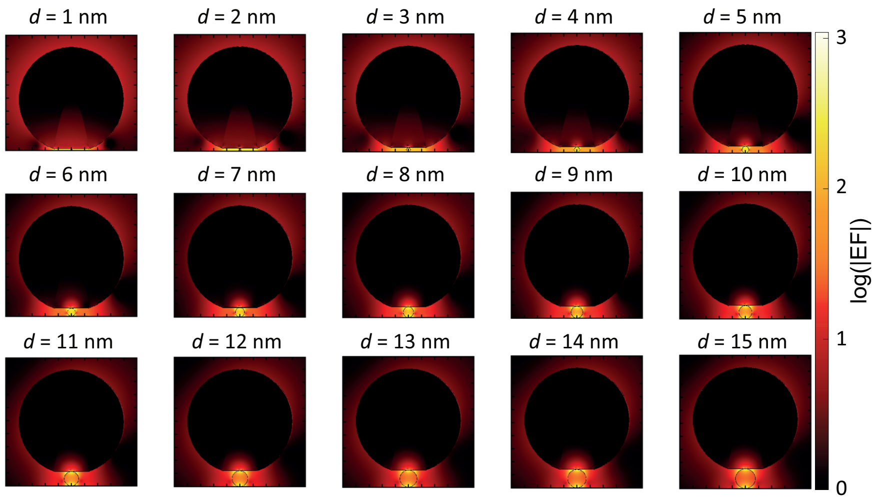

Supplementary Fig. 15 Electric field distribution for nano-object inside the nanocavity with D = 80 nm and d = 1 nm to 15 nm.

We calculated the electric field distribution within a nanocavity containing a nano-object (Supplementary Fig. 15). For very small gaps (1 to 3 nm), the electric field is less tightly confined and spreads across the entire gap region. As the diameter of the nano-object increases from 1 nm to 15 nm, the electric field becomes increasingly concentrated around the nano-object within the gap. The nano-object effectively

acts as a hotspot, strongly localizing and enhancing the electromagnetic field. This pronounced field confinement leads to a significant increase in the nano-object's scattering cross-section, similar to the mechanisms observed in surface-enhanced Raman scattering (SERS). The strong near-field coupling observed in these configurations highlights their potential for applications in optical sensing, single-molecule detection, and various forms of enhanced spectroscopy [29].

SI. N Sample fabrication process

In the method section, we discuss the sample fabrication process, and we elaborate further on the procedure here. We prepared the Au substrate using template stripping by thermally depositing a 100 nm Au layer on a silicon wafer. We apply UV-curable epoxy, place glass coverslips on top, and cure under UV light for 20 minutes (Supplementary Fig. 16). The glass-bound Au surfaces were stored and peeled off when needed. We functionalized the nano-objects with cysteamine, which binds to Au via a thiol group and provides a positively charged amino group for electrostatic interaction. We dried the functionalized nano-object onto the Au substrate at a density of 1600 particles/μm^2. Then we drop-cast the nanoprobe solution, optimized a 120-second deposition to form single nanocavities, and rinsed the sample to remove unbound probes.

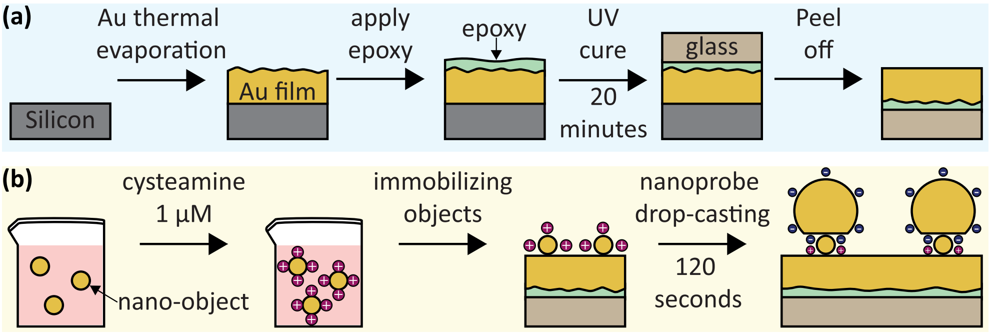

Supplementary Fig. 16 Sample fabrication process. **(a)** Template stripping process: Au film is deposited onto a silicon wafer; a UV-curable epoxy is applied to the Au surface; a glass coverslip is placed atop the epoxy; the sample is cured using UV light; and the coverslip is peeled off to retrieve the smooth Au surface. **(b)** nanocavity assembly process: functionalizing the nano-objects using cysteamine hydrochloride; immobilizing the nano-object on the surface by drying the solution of the nano-object on the Au film; drop casting functionalized nanoprobes, and forming a nanocavity, due to the electrostatic attraction of the nano-object and nanoprobe.

SI. O Concentration of cysteamine

To obtain the optimal concentration of the cysteamine to functionalize the nano-object, we measure the UV-Vis absorbance of the nano-object colloidal solution

functionalized with different concentrations of the cysteamine from 0 μM to 20 μM (Supplementary Fig. 17a). The absorbance spectrum of the colloidal nano-object changes significantly at different cysteamine concentrations. As the cysteamine concentration increases, the absorbance intensity decreases at 518 nm, and a new peak appears at 680 nm. Results indicate that a concentration of 1μM is the optimal concentration of cysteamine due to minimal changes in the absorbance ratio at 680 nm and 518 nm (Supplementary Fig. 17b).

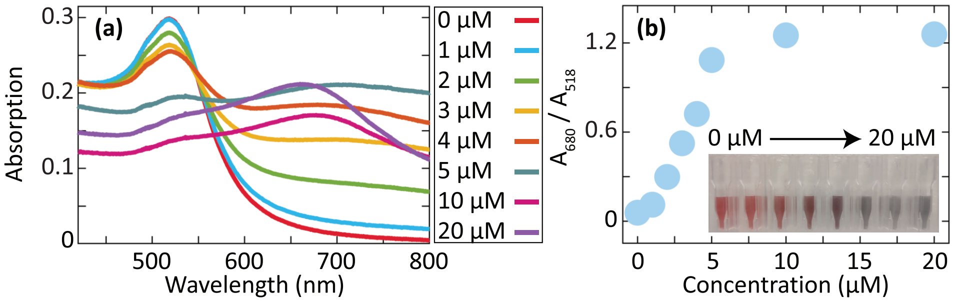

Supplementary Fig. 17 **(a)** UV-vis absorbance spectra of 10 nm nano-object functionalized with different concentrations of cysteamine varying from 0 μM to 20 μM. **(b)** Absorption ratio at 680 nm and 518 nm as a function of cysteamine concentration, determined from the UV-vis absorbance spectra. Inset is the corresponding photo of the nano-object colloidal solution functionalized with different concentrations of the cysteamine.

References

[1] Economou, E. Surface plasmons in thin films. *Physical review* **182**, 539 (1969).

[2] Tserkezis, C. *et al.* Hybridization of plasmonic antenna and cavity modes: Extreme optics of nanoparticle-on-mirror nanogaps. *Physical Review A* **92**, 053811 (2015).

[3] Baumberg, J. J., Aizpurua, J., Mikkelsen, M. H. & Smith, D. R. Extreme nanophotonics from ultrathin metallic gaps. *Nature materials* **18**, 668–678 (2019).

[4] Li, G.-C., Zhang, Q., Maier, S. A. & Lei, D. Plasmonic particle-on-film nanocavities: a versatile platform for plasmon-enhanced spectroscopy and photochemistry. *Nanophotonics* **7**, 1865–1889 (2018).

[5] Hendriks, A. L. *et al.* Detecting single nanoparticles using fiber-tip nanophotonics. *Optica* **11**, 512–518 (2024).

[6] Liang, F. & Quan, Q. Detecting single gold nanoparticles (1.8 nm) with ultrahigh-q air-mode photonic crystal nanobeam cavities. *Acs Photonics* **2**, 1692–1697 (2015).

[7] Su, J., Goldberg, A. F. & Stoltz, B. M. Label-free detection of single nanoparticles and biological molecules using microtoroid optical resonators. *Light: Science & Applications* **5**, e16001–e16001 (2016).

[8] Armani, A. M., Kulkarni, R. P., Fraser, S. E., Flagan, R. C. & Vahala, K. J. Label-free, single-molecule detection with optical microcavities. *science* **317**, 783–787 (2007).

[9] Zhao, Y. & Liu, F. Multi-target detection and sizing of single nanoparticles using an optical star polygon microcavity. *Optics Express* **31**, 29051–29060 (2023).

[10] Jain, P. K., Huang, W. & El-Sayed, M. A. On the universal scaling behavior of the distance decay of plasmon coupling in metal nanoparticle pairs: a plasmon ruler equation. *Nano letters* **7**, 2080–2088 (2007).

[11] Dolinnyi, A. I. Nanometric rulers based on plasmon coupling in pairs of gold nanoparticles. *The Journal of Physical Chemistry C* **119**, 4990–5001 (2015).

[12] Zhang, W., Li, Q. & Qiu, M. A plasmon ruler based on nanoscale photothermal effect. *Optics Express* **21**, 172–181 (2013).

[13] Jain, P. K. & El-Sayed, M. A. Surface plasmon coupling and its universal size scaling in metal nanostructures of complex geometry: elongated particle pairs and nanosphere trimers. *The Journal of Physical Chemistry C* **112**, 4954–4960 (2008).

[14] Griffiths, D. J. *Introduction to electrodynamics* (Cambridge University Press, 2023).

[15] Benz, F. *et al.* Generalized circuit model for coupled plasmonic systems. *Optics Express* **23**, 33255–33269 (2015).

[16] Jain, P. K., Eustis, S. & El-Sayed, M. A. Plasmon coupling in nanorod assemblies: optical absorption, discrete dipole approximation simulation, and exciton-coupling model. *The Journal of Physical Chemistry B* **110**, 18243–18253 (2006).

[17] Gluodenis, M. & Foss, C. A. The effect of mutual orientation on the spectra of metal nanoparticle rod- rod and rod- sphere pairs. *The Journal of Physical Chemistry B* **106**, 9484–9489 (2002).

[18] Bohren, C. F. & Huffman, D. R. *Absorption and Scattering of Light by Small Particles* (John Wiley & Sons, New Jersey, USA, 1998).

[19] Geisler, M. *et al.* Single-crystalline gold nanodisks on ws2 mono-and multilayers for strong coupling at room temperature. *Acs Photonics* **6**, 994–1001 (2019).

[20] Kolwas, K. Optimization of coherent dynamics of localized surface plasmons in gold and silver nanospheres; large size effects. *Materials* **16**, 1801 (2023).

[21] Zeman, E. J. & Schatz, G. C. An accurate electromagnetic theory study of surface enhancement factors for silver, gold, copper, lithium, sodium, aluminum, gallium, indium, zinc, and cadmium. *Journal of Physical Chemistry* **91**, 634–643 (1987).

[22] Yang, H. U. *et al.* Optical dielectric function of silver. *Physical Review B* **91**, 235137 (2015).

[23] Mertens, J. *et al.* Tracking optical welding through groove modes in plasmonic nanocavities. *Nano Letters* **16**, 5605–5611 (2016).

[24] Yang, D.-J. *et al.* Analytical analysis of spectral sensitivity of plasmon resonances in a nanocavity. *Nanoscale* **11**, 10977–10983 (2019).

[25] Ordal, M. A., Bell, R. J., Alexander, R. W., Long, L. L. & Querry, M. R. Optical properties of fourteen metals in the infrared and far infrared: Al, co, cu, au, fe, pb, mo, ni, pd, pt, ag, ti, v, and w. *Applied optics* **24**, 4493–4499 (1985).

[26] Smith, D., Shiles, E., Inokuti, M. & Palik, E. Handbook of optical constants of solids. *Handbook of optical constants of solids* **1**, 369–406 (1985).

[27] Chikkaraddy, R. *et al.* Single-molecule strong coupling at room temperature in plasmonic nanocavities. *Nature* **535**, 127–130 (2016).

[28] Novotny, L. & Hecht, B. *Principles of nano-optics* (Cambridge university press, 2012).

[29] Liu, L., Bhaskar, S. & Cunningham, B. T. Hybrid interfacial cryosoret nano-engineering in photonic resonator interferometric scattering microscopy: Insights from nanoparticles and nano-assemblies. *Applied Physics Letters* **124** (2024).
